# Comparison of Auscultation Quality Using Contemporary Digital Stethoscopes

**DOI:** 10.2196/54746

**Published:** 2024-11-08

**Authors:** Norah Saraya, Jonathon McBride, Karandeep Singh, Omar Sohail, Porag Jeet Das

**Affiliations:** 1 Department of Learning Health Sciences University of Michigan Ann Arbor, MI United States

**Keywords:** auscultation, digital stethoscopes, valvular heart disease

## Introduction

Auscultation is a common clinical tool for assessing patients for valvular heart disease (VHD). Its accuracy for screening varies based on operator’s training and experience. While echocardiography remains the gold standard in diagnosing VHD due to its greater accuracy, especially among individuals with obesity [1,2], its use is cost-prohibitive for screening.

Digital stethoscopes have emerged as a more cost-effective alternative [3]. Digital audio collection through a stethoscope has enabled automated diagnosis of VHD and may reduce interoperator variability. While prior work has not found large differences between digital stethoscopes [4], the poor audio quality in older digital stethoscopes [5] coupled with recent advances in audio processing have led to questions as to whether more recent digital stethoscopes may provide a viable alternative to echocardiography in the diagnosis of VHD.

In this study, we compare two contemporary digital stethoscopes, the Eko DUO and 3M Littmann CORE, in their sound quality with both bedside and recorded sounds.

## Methods

### Overview

Heart sounds were collected from four anatomical locations for 25 patients within a university hospital in Ann Arbor, MI. Participants were eligible if they were 18 years or older and had undergone or planned to undergo transthoracic echocardiography within 7 days of enrollment. Informed consent was obtained. Each stethoscope was used to obtain a phonocardiogram recording at the aortic and pulmonary (ie, second intercostal space along the right and left sternal border, respectively), tricuspid (fifth intercostal space along the left sternal border), and mitral valve (fifth intercostal space at the midclavicular line) for 30 seconds each. Sound quality and the presence of a murmur were assessed by a trained study team member (NS) at the bedside using the stethoscope and separately during playback of the recorded sounds using headphones as described previously [5]. Both stethoscopes were used to collect sounds for each patient, alternating which one was used first.

Four components of auscultation were assessed, whether (1) S1 was detected, (2) S2 was detected, (3) a murmur was detected, and (4) there was confidence in the assessment (yes or no).

### Ethical Considerations

Our study was reviewed and approved by the University of Michigan institutional review board (HUM00133770). Study participants consented to participate in the study. Data were stored with coded identifiers with access to medical record numbers to facilitate chart review. Participants were not financially compensated.

## Results

The mean age of the 25 participants was 65 (SD 11.78) years, 11 (44%) were women, and 2 (8%) were Black. The mean BMI was 34.3 (SD 7.98) kg/m^2^, and congestive heart failure was present in 6 (24%) participants, hypertension in 17 (68%) participants, and valvular disease in 1 (4%) participant. We performed 400 evaluations (25 patients × 4 locations × 2 contexts, live and recorded).

Compared to the Eko DUO stethoscope, the 3M Littmann CORE stethoscope performed worse in the ability to hear S1 (odds ratio 0.56, 95% CI 0.33-0.95) and appreciate murmurs (odds ratio 0.32, 95% CI 0.21-0.50; Table 1). The ability to hear S2 and confidence level were not different. As compared to live auscultation, recorded sounds were not different across all four evaluation measures (Table 2).

We performed a sensitivity analysis to explore the patient-level findings for the stethoscope comparisons due to the large effect size. The proportion of variance explained by the random intercept (patient identifier) was 33% for the detection of S1, 22% for the detection of S2, 61% for the presence of murmurs, and 12% for confidence. After accounting for the random intercept, the odds ratio between stethoscopes (with Eko DUO stethoscope as the reference group) for hearing S1 was 0.54 (95% CI 0.31-0.93; *P*=.03), for hearing S2 was 0.62 (95% CI 0.37-1.04; *P*=.07), for the presence of a murmur was 0.23 (95% CI 0.14-0.39; *P*<.001), and for confidence was 0.98 (95% CI 0.66-1.46; *P*=.93).

**Table 1:** Comparison of findings between the Littmann CORE and Eko DUO stethoscopes^a^.

Measure	Littmann CORE (n=200)	Eko DUO (n=200)	Littmann stethoscope (with Eko DUO as reference), odds ratio (95% CI)	*P* value
Ability to hear S1 (%)	79	87	0.56 (0.33-0.95)	.04
Ability to hear S2 (%)	78	85	0.63 (0.38-1.04)	.08
Murmur (%)	20	44	0.32 (0.21-0.50)	<.001
Confidence (%)	50	51	0.98 (0.66-1.45)	.92

^a^For each stethoscope, we included evaluations collected from both live and recorded sounds across all anatomic locations.

**Table 2:** Comparison of findings assessed during live auscultation and on recorded sounds^a^.

Measure	During live auscultation (n=200)	Based on recorded sounds (n=200)	Recording (with live auscultation as reference), odds ratio (95% CI)	*P* value
Ability to hear S1 (%)	85	81	0.75 (0.33-1.27)	.29
Ability to hear S2 (%)	83	79	0.77 (0.46-1.27)	.31
Murmur (%)	32	32	0.98 (0.64-1.49)	.91
Confidence (%)	52	50	0.94 (0.64-1.40)	.76

^a^For live and recorded sounds, we included evaluations collected from both stethoscopes across all anatomic locations.

## Discussion

The results suggest that there are potentially meaningful differences in sound quality among contemporary stethoscopes. While both stethoscopes incorporate technology from Eko, the Eko DUO stethoscope appeared to perform better in the ability to detect S1 and murmurs; the point estimate for 3M Littmann CORE in detecting S2 was 0.63, but this was not statistically significant (*P*=.08). We did not find statistically significant differences across live versus recorded sounds.

Our study is limited by a small sample size drawn from a single hospital. However, our use of a consistent rating process applied to multiple contexts and anatomic locations provides evidence that all digital stethoscopes may not be created equal.

## Abbreviations

VHD: valvular heart disease

## Data Availability

Our data are not publicly available.
